# Monthly Summary

**Published:** 1897-11

**Authors:** 


### Monthly Summary.

---

THE ARREST OF HEMORRHAGE, even from slight wounds, in patients affected by hemophilia; is sometimes a serious problem, with not rarely a fatal solution. Beinwald has recently adopted a new plan in the case of a child, aged two years, the subject of hemophilia. Having failed to arrest the hemorrhage from a small wound on the face by the application of perchloride of iron, he obtained some blood by aspiration from a healthy subject and deposited it on the wound. In a few minutes it coagulated, and the hemorrhage at once ceased. His explanation of the action of the remedy is that it supplies the ferment necessary for thrombosis in the small vessels. Whether this is correct or not it is impossible to say in the absence of definite knowledge of the pathology of hemophilia. As affording his explanation some support may be mentioned the success obtained by Wright in his experiments with a solution of fibrin ferment and chloride of calcium as a styptic. Bienwald's ingenious method certainly deserves further trial.—*American Journal of Surgery.*

---

PECULIARITIES OF WOMAN.—Women pin from left to right, men from right to left. Women button from right to left, men from left to right. Women stir from left to right (their tea, for instance), men from right to left. Women seldom know the difference between a right and left shoe, and if a housemaid brings up a man's boots, she will, nine times out of ten, place them so that the points will diverge. London *Truth* inquires whether these peculiarities can be explained?

DON'T USE PLAIN TEETH in constructing artificial dentures where the rubber, either pink, red or black, will show. If you have difficulty in making perfect joints with gum sections, then follow these directions—viz. ; Grind the joints upon a large, true-running corundum wheel, making the fitting close front and back. Before investing, cover the front surface with cement and allow one hour to harden. Let the case, after investing, remain over night before packing, so that the plaster will become thoroughly set, and then proceed to pack without a surplus of rubber. If it is not possible to let the case remain over night before packing, then grind the inner half of the joints to a V shape, and fill with oxychloride of zinc, allowing one hour for hardening before packing.—*Western Dental Journal.*

---

SINS.—By Dr. Emory Laughlin.—First Sin—Operating on Hopeless Cases.—There are some—and they are called surgeons—who will undertake almost any kind of an operation, regardless of the prospects of cure or failure, nay, even of life or death; and sometimes without consideration of the question of their ability to do the work properly.

The incentives are ; (a) to get a good fee; (b) to have the credit of operating, especially in rare or difficult cases; (c) to obtain control of the influential patient from a conservative physician, who, recognizing the hopelessness of the case, is doing all he can to ameliorate suffering and prolong life, but persistently refuses entreaties of patient and friends to 'do something'; (d) ignorance of real condition.

Second Sin—Pretending to be Clean, but Failing.—Every one now knows the inestimable value of asepsis; most surgeons pretend to practice it; nearly all failed. The clean (ideally, surgically, truly clean) surgeons of America do not number a dozen ! I know them—I mean the leaders of the profession, not the hewers of wood and tillers of soil, not the common, every-day workers like you and me, but the men to whom we look for guidance. One point of failure is dirty finger nails. Lawrence Tait's remark, that 'The high mortality rate in abdominal surgery found in the work of German surgeons is easily accounted for—they don't clean their finger nails,' is applicable to the methods of some surgeons not Teutonic.

Third Sin—Undercharging, in Order to Secure an Operation.—That is a sin too often committed by men who stand high in the profession; and this assertion is no idle figment of the imagination, either.

Fourth Sin—Stealing Patients from Other Surgeons.—This is more often the cause of hard feelings between surgeons than any other one thing, though, as it is done usually in such a genteel, 'ethical' way, there is no recourse left to the injured party, except to growl.—*Journal of Surgery.*

---

THE NEW DENTAL LAW in Pennsylvania has this wise provision, viz: "Each applicant shall also furnish to the board satisfactory evidence of his or her proficiency in the manipulative procedures of dentistry, either by producing an example of his or her work, duly attested by the demonstrator in charge of the clinic of the college issuing his or her diploma, or by a practical demonstration of his or her skill in the presence of the examining members of said board."

---

LACTIC ACID IN PYORRHŒA.—Dr. W. J. Younger says that the excoriation of the mouth and gums may be prevented by the application of the oleo-stearate of zinc to exposed surfaces which it is desired to protect against its action, when lactic acid is used in pyorrhœa.

---

THE DENTAL STUDENT who is neat, clean, and well-behaved, who gives respect to his teachers and their teaching, is the man who will win success, and upon whom the future success of the profession must always depend.

The lady was a neutral in politics, but not a bit behind in her knowledge of the issues of the late campaign. She remarked : "Doctor, if you'll not regard it dictatorial on my part, I'd suggest that you McKinleyize this tooth and merely Bryanize the molar. The latter's out of sight, you know, and no one will ever be the wiser."

"Beg pardon ?"

"You mean you don't understand me ? Why, crown this tooth with gold, and transfix that one with silver,—amalgam I think some of you gentlemen call it."—"Odontographic Journal."

---

Small boy patient : "Doctor, do you know the commandments ?"

Doctor . "Yes."

"Repeat 'em."

Doctor repeats.

"What's the other ?"

"There is no other."

"Yes "

"What is it ?"

The 11th : "Thou shalt not rubber stretch thy neck."

---

SAID a patient who had worn an arsenical dressing several hours with great discomfort and returned for at least temporary relief : "Doctor, that tooth aches like the devil."

"That's right."

"Oh, is it ?"

"Yes."

"Thanks! I felt a bit anxious about it; good-day."—*Odontographic Journal.*

---

A NEW WART CURE.—Chromic acid, one hundred grains to the ounce, applied frequently with a tooth-pick, will remove small warts or similar growths.

DIAMOND DRILLS, HOW TO USE.—A writer in *Ash's Quarterly* says: "In using a diamond drill for making cavities in artificial teeth, it is most important to bear in mind that the point should be dipped in sweet oil or water, *and placed on the work before the engine is started.* The engine should be stopped as often as the drill shows signs of becoming dry, and each time after it has been dipped in the lubricant it must again be placed on the work before the engine is started. It is, perhaps, needless to add that the hand-piece must be held firmly and steaily in position to insure success and to avoid breaking the drill."—*Catching's Dental Weekly.*

---

TO CLEAN and otherwise improve the working qualities of an oilstone, smear a flat block of wood with glycerine and fine pumice and rub the stone, face down, till all traces of previous usage have disappeared. To ruin an oil stone, clean it with kerosene. Its hardness will then rival Pharoh's nipples.—"Odontographic Journal."

---

TO KEEP SPITTOON CLEAN.—Mr. A. W. Wright, Jr., of London, finds that a little sulphate of copper sprinkled inside the spittoon (metal or earthenware) before the day's operations commence, prevents any unpleasant odor, does not allow the interior of the spittoon to become furred, and renders it much easier to cleanse than usual.—"Ash's Circular."

---

IT is a mistake to begin to prepare and fill teeth before removing salivary calculus and stains. No operator can tell the requirements of a case till the surfaces of the teeth are bright and free from discoloration, so that the line of demarkation between the normal and the diseased tissue may be clearly observed.--T. W. Brophy, in "Review."

